# Inhibition of METTL3 Attenuates Renal Fibrosis by Upregulating ABCG2 m6A Modifications via IGF2BP2‐Dependent Mechanisms in Hyperuricemic Nephropathy

**DOI:** 10.1111/jcmm.70468

**Published:** 2025-03-18

**Authors:** Tong Zu, Hang Yang, Jie Wang, Shuangjian Li, Yue Yu, Kuo Zhang, Xiuxiu Song, Jie Ying, Yaru Yang, Xian Wang, Juan Jin

**Affiliations:** ^1^ School of Basic Medicine, School of Pharmacy Anhui Medical University Hefei China; ^2^ Inflammation and Immune Mediated Diseases Laboratory of Anhui Province, Anhui Institute of Innovative Drugs, School of Pharmacy Anhui Medical University Hefei Anhui China; ^3^ Department of Clinical Pharmacology The Second Affiliated Hospital of Anhui Medical University Hefei Anhui China; ^4^ Department of Nephrology Fuyang People's Hospital of Anhui Medical University Fuyang Anhui China

**Keywords:** ABCG2, hyperuricemia, IGF2BP2, METTL3, mRNA m6A

## Abstract

Hyperuricemia has been linked to kidney problems including hyperuricemic nephropathy (HN), which is characterised by inflammation and fibrosis in the kidneys. HN is frequently observed in patients with chronic gout. However, the causes of HN are not fully understood and effective treatments are limited. The status of RNA m6A, expression, and location of METTL3 in the kidney was evaluated in mice with HN. The mechanism of the METTL3‐associated ABCG2 downregulation was further studied in mTEC cells and a potassium oxazinate + adenine‐induced mice model and adeno‐associated virus 9 (AAV9)‐mediated METTL3 silencing mice. Expressions of ABCG2, α‐SMA, collagen‐1, TGF‐β1, IL‐1β, IL‐6, and TNF‐α were analysed using real‐time PCR and western blotting. Hyperuricemia led to elevated m6A levels and METTL3 expression in mouse kidneys. METTL3 was mainly located in mTEC cells. METTL3‐specific inhibitor STM2457 alleviated uric acid‐induced inflammatory and fibrotic responses in mTEC cells. Mechanistically, ABCG2 was identified as a target of METTL3 by RNA sequencing. The stability of ABCG2 was decreased through the binding of IGF2BP2 (insulin‐like growth factor 2 binding protein 2) to its m6A‐modified stop codon regions. Silencing or inhibition of METTL3 significantly reduced uric acid‐induced cell injury and increased ABCG2 expression, leading to uric acid excretion. In vivo data showed that AAV9‐mediated METTL3 silencing significantly alleviated renal dysfunction and fibrosis in HN mice. Our study provides the first evidence that METTL3 regulates uric acid excretion by controlling the m6A levels of ABCG2 through the binding of IGF2BP2, and inhibiting METTL3 can effectively alleviate kidney damage caused by hyperuricemia, showing potential as a therapy for HN.

## 1 Introduction

Hyperuricemia is closely correlated with an increased risk of cardiovascular disease, hypertension, chronic kidney disease (CKD), and kidney failure [1]. Hyperuricemic nephropathy (HN) is a common clinical complication of hyperuricemia, which leads to crystalline kidney stones, chronic interstitial nephritis, and renal fibrosis [2, 3]. Some clinical trials indicated that urate‐lowering therapy improved kidney function [4, 5]. However, whether lowering serum urate pharmacologically mitigates the risk of progressive HN remains uncertain.

Epigenetic modifications, without changing DNA sequences, involve the onset and progression of kidney diseases through methods like RNA interference, histone modification, and DNA methylation [6]. Among these modifications, m6A, a common RNA methylation, participates in post‐transcriptional RNA metabolism, regulating reversible modifications, alternative splicing, stability, and translation [7, 8]. It plays a crucial role in processes such as cell differentiation, development, and metabolism [9]. m6A methylation is catalysed by multi‐component methyltransferase complexes, including METTL3 and METTL14 [6]. METTL3‐mediated m6A modifications in various target genes have been linked to several diseases, drawing recent attention to its role in kidney diseases [10, 11]. In a previous study, we showed that DNA methylation of FTO induced the m6A methylation of adenosines in peroxisome proliferator‐activated receptor α, which contributed to alcohol‐induced kidney injury [12]. Recently, we found that METTL3 promoted m6A modifications of TAB3, while both genetic and pharmacological inhibition of METTL3 attenuated cisplatin‐induced renal injury and inflammation [13]. Zhou et al. indicated that METTL3 promoted apoptosis in renal tubular epithelial cells in cisplatin‐induced acute renal injury [14]. Additionally, METTL3‐mediated methylation of MALAT1 m6A exacerbates renal fibrosis in obstructive nephropathy [15]. Despite these findings, the function of m6A modifications in HN is largely unknown.

Urate excretion from the human kidney is tightly regulated by reabsorption and excretion [16]. According to previous research results, urate transports including OAT1–4, URAT1, GLUT9, ATP‐binding cassette subfamily G member 2 (ABCG2) are closely related to the serum uric acid level [17]. Among them, ABCG2 is important in uric acid excretion to transport urate out of the cells into the renal tubular lumen, and its transport function is coupled to energy from ATP hydrolysis [18]. As one of the major urate transporters in the kidney, ABCG2 dysfunction is a risk factor for HUA [19]. Dysfunction of ABCG2 is a common mechanism of hyperuricemia [20], while upregulation of ABCG2 could alleviate HN [21, 22, 23]. Therefore, ABCG2 may play crucial functions in the pathophysiological processes of HN.

We found that METTL3 was consistently induced in murine models of HN and uric acid‐treated renal tubular epithelial cells (TECs), which show an obvious correlation with renal fibrosis and injury. Therefore, we hypothesised that METTL3 may act as a regulator of high uric acid‐induced renal fibrosis. We tested our hypothesis in vivo in AAV9‐mediated METTL3 silencing mice, and in vitro in METTL3‐silenced TECs, the downstream target of METTL3 was also identified. The results indicate that METTL3 inhibitors may be a potential treatment for HN.

## 2 Materials and Methods

### 2.1 Study Design

In this study, an HN model was established in C57BL/6 mice through continuous administration of potassium oxyazinate and adenine for 21 days. Mice in both the normal group and the HN group were monitored for weight, kidney appearance, and various blood parameters, including uric acid, creatinine, and urea nitrogen, to confirm the successful construction of the model. The methylation of m6A in total mRNA from mice was examined to investigate the role of m6A methylation in hyperuricemic nephropathy. Additionally, changes in METTL3 levels were assessed. METTL3 gene interference was performed using adeno‐associated virus injected into the mice's tail vein to knock down METTL3 levels. The RNA‐seq technique was employed to identify differential genes, and the SRAMP database was utilised to predict m6A modification sites on relevant differential genes under HN conditions. Furthermore, in vitro gene silencing and specific inhibitors were used to explore the role and specific mechanisms of METTL3 in HN, providing novel insights into the pathogenesis and potential therapeutic targets of HN.

### 2.2 Reagents and Materials

The reagents and materials used include: anti‐METTL3 antibody at a dilution of 1:1000 (Wanleibio, Cat# WL05194), anti‐collagen‐1 antibody at a dilution of 1:1000 (Wanleibio, Cat# WL0088), anti‐α‐SMA antibody at a dilution of 1:500 (Wanleibio, Cat# WL02510), goat anti‐mouse IgG antibody at a dilution of 1:1000 (Affinity, Cat# S0002), goat anti‐rabbit IgG antibody at a dilution of 1:1000 (Affinity, Cat# S0001), anti‐IGF2BP2 antibody at a dilution of 1:2000 (Proteintech, Cat# 11601‐1‐AP), anti‐ABCG2 antibody at a dilution of 1:1000 (Affinity, Cat# AF5177), anti‐β‐actin antibody at a dilution of 1:1000 (Affinity, Cat# AF7018), anti‐Kim1 antibody at a dilution of 1:1000 (Affinity, Cat# DF12798), and BCA protein concentration assay kit (Beyotime, Cat# P0006), Haematoxylin and eosin (H&E) staining kit (Solarbio, Cat#G1120), Masson's trichrome staining kit (Solarbio, Cat# G1340), Sirius red staining solution (Solarbio, Cat# G1472), creatinine (Cr) assay kit (Jiancheng, Cat# C011‐2‐1), serum uric acid assay kit (Jiancheng, Cat# C012‐2‐1), blood urea nitrogen (BUN) assay kit (Jiancheng, Cat# C013‐1‐1), and *Lotus tetragonolobus* lectin (LTL) (Vector Laboratories, Cat# FL‐1321‐2). Mouse METTL3 gene interference adeno‐associated virus was purchased from Hanbio (Shanghai, China).

### 2.3 Animals

Male C57BL/6J mice (6–8 weeks old, approximately 20–23 g) were obtained from Hangzhou Ziyuan Laboratory Animal Technology Co. Ltd. All animal procedures were ethically approved by the Animal Experimentation Ethics Committee of Anhui Medical University. Mice were housed under standard conditions with 12 h of daylight, relative humidity between 40% and 65%, and room temperature maintained at approximately 20°C–23°C.

### 2.4 Animal Model

Upon purchase, mice underwent a 7‐day adaptation period before being randomly divided into the normal group and the high uric acid nephropathy group. Mice in the HN group were daily administered potassium oxazinate (Macklin, Cat# P831461) (250 mg/kg) + adenine (Biofrox, Cat# 1163GR005) (100 mg/kg). The normal group received an equivalent volume of sodium carboxymethyl cellulose solution (CMC‐Na) (Sigmaaldrich, Cat# C5678) for 21 days. On the 21st day, blood and kidney samples were collected for analysis.

### 2.5 AAV‐9‐Induced METTL3 Silencing Mice

The interference sequence of the adeno‐associated virus targeting the Mettl3 gene is GGACCAAGGAAGAGTGCAT. Upon purchase, mice underwent a 7‐day adaptation period. Adeno‐associated virus‐mediated knockdown of the METTL3 gene in mice was developed and supplied by Hanheng Biotechnology in Shanghai, China. 100 μL of pHBAAV‐METTL3 and the vector (1 × 10^12^ virus genomes per millilitre) were injected into the tail vein of mice using a 31‐gauge needle. Lentiviral vectors carrying an empty vector (EV) plasmid were purchased from Hanheng Biotechnology in Shanghai, China. The mice were divided into four groups: (i) mice injected with the EV plasmid, (ii) mice injected with the pHBAAV‐METTL3 plasmid, (iii) mice injected with the pHBAAV‐METTL3 plasmid and induced with HN, and (iv) mice injected with the EV plasmid and induced with HN. The mice received control lentiviral expression vectors via tail vein injection. 4 weeks later, mice in the HN group were daily administered potassium oxazinate (250 mg/kg) + adenine (100 mg/kg). The normal group received an equivalent volume of sodium CMC‐NA for 21 days. On the 21st day, blood and kidney samples were collected for analysis.

### 2.6 Cell Culture and Model Establishment

Mouse tubular epithelial cells (mTECs) were provided by Prof. Hui‐yao Lan (Chinese University of Hong Kong). mTECs were cultured in DMEM/F12 medium supplemented with 10% FBS at 37°C with 5% CO_2_ in the incubator. mTECs were stimulated with 15 mg/dL of uric acid after 2 h of starvation.

### 2.7 Reverse Transcription Polymerase Chain Reaction

Total RNA from cells and kidney tissues was extracted using Trizol according to the manufacturer's instructions. Reverse transcription polymerase chain reaction (RT‐PCR) analysis was performed as previously described. All reaction reagents were obtained from TAKARA, Japan. The mRNA levels were normalised to β‐actin using the primers listed in Table 1.

**TABLE 1:** Primer sequences used for real‐time polymerase chain reaction (PCR).

Genes (mouse)	Forward primer (5′–3′)	Reverse primer (5′–3′)
β‐Actin	CATTGCTGACAGGATGCAGAA	ATGGTGCTAGGAGCCAGAGC
α‐SMA	GCACCTGGATCATTGCTTCC	TCCTTGGAAGTACTGCCGTT
TGF‐β1	GGACTCTCCACCTGCAAGAC	GACTGGCGAGCCTTAGTTTG
TNF‐α	CATCTTCTCAAAATTCGAGTGACAA	TGGAGTAGACAAGGTACAACCC
IL‐1β	GAAATGCCACCTTTTGACAGTG	TGGATGCTCTCATCAGGACAG
ABCG2	TCTGTCTTCCTGGTCCTCTC	TTGAAATGGGCAGGTTGAGG
METTL3	GTGCAGCCCAACTGGATTACT	AGTCCTCTTAAGGTGTGGCCT
METTL14	CGAAGCTGGGGCATGGATA	AGATGTATCATAGGAAGCCCTGC
ALKBH5	TTCCAGTTCAAGCCCATCCG	CGGTGCATCTAATCTTGTCTTCC
FTO	TGGCGACGTCTCGTTGAAAT	GCACCGCATTTGTCATGCT
IGF2BP1	CGGCAACCTCAACGAGAGT	GTAGCCGGATTTGACCAAGAA
IGF2BP2	GACTACCCCGACCAGAACTG	GAGGCGGGATGTTCCGAATC
IGF2BP3	CCTGGTGAAGACGGGCTAC	TACACTTCCATCGGTTTCCCA

### 2.8 Western Blot Analysis

Protein lysates from kidney tissues and cells were extracted following standard protocols. Briefly, proteins were electrophoresed on a 10% SDS‐PAGE gel and then transferred to PVDF membranes. After blocking nonspecific binding with 5% milk for 1 h, the membranes were incubated overnight at 4°C with primary antibodies, including METTL3 (Wanleibio, Cat# WL05194), Col‐1 (Wanleibio, Cat# WL0088), Kim1 (Affinity, Cat# DF12798), ABCG2 (Affinity, Cat# AF5177), α‐SMA (Wanleibio, Cat# WL02510), IGF2BP2 (Proteintech, Cat# 11601‐1‐AP), and β‐actin (Affinity, Cat# AF7018). Subsequently, membranes were incubated with secondary antibodies for 1.5 h at room temperature. Images were captured using the Licor/Odyssey infrared imaging system, and quantitative analysis was performed using ImageJ software.

### 2.9 Renal Histology

Paraffin‐embedded kidney sections (4 μm) were prepared using routine procedures. Histological damage was assessed through HE, Sirius Red, and Masson staining according to the assay kits' instructions.

### 2.10 Renal and Liver Function Detection

Blood collected from mice after anaesthesia was centrifuged at 3000 rpm for 20 min at 4°C to obtain serum, which was then used to measure Cr, BUN, and UA levels using assay kits from Nanjing, China.

### 2.11 Immunofluorescence

Cells were fixed in acetone, blocked in 10% BSA for 30 min, and incubated overnight with primary antibodies (METTL3 or ABCG2). Afterward, the cells were incubated with a secondary antibody (goat anti‐rabbit IgG‐rhodamine) for 1.5 h and stained with DAPI for 10 min. Images were visualised and captured using fluorescence microscopy.

### 2.12 RNA Stability

To assess ABCG2 stability, cells were incubated with actinomycin (5 μg/mL) to terminate transcription. Samples were collected at 0, 3, and 6 h after termination. The total RNA was extracted, and the ABCG2 level was determined by real‐time PCR.

### 2.13 Statistical Analysis

Quantitative data were presented as mean ± S.E.M. from three independent in vitro experiments or six mice in vivo. Statistical analyses were performed using independent sample *t*‐tests and one‐way ANOVA followed by Newman–Keuls post hoc test to examine differences in means among different groups. Statistical significance was set at *p* < 0.05.

## 3 Results

### 3.1 METTL3 Expression is Highly Induced in HN Mice

**FIGURE 1:**
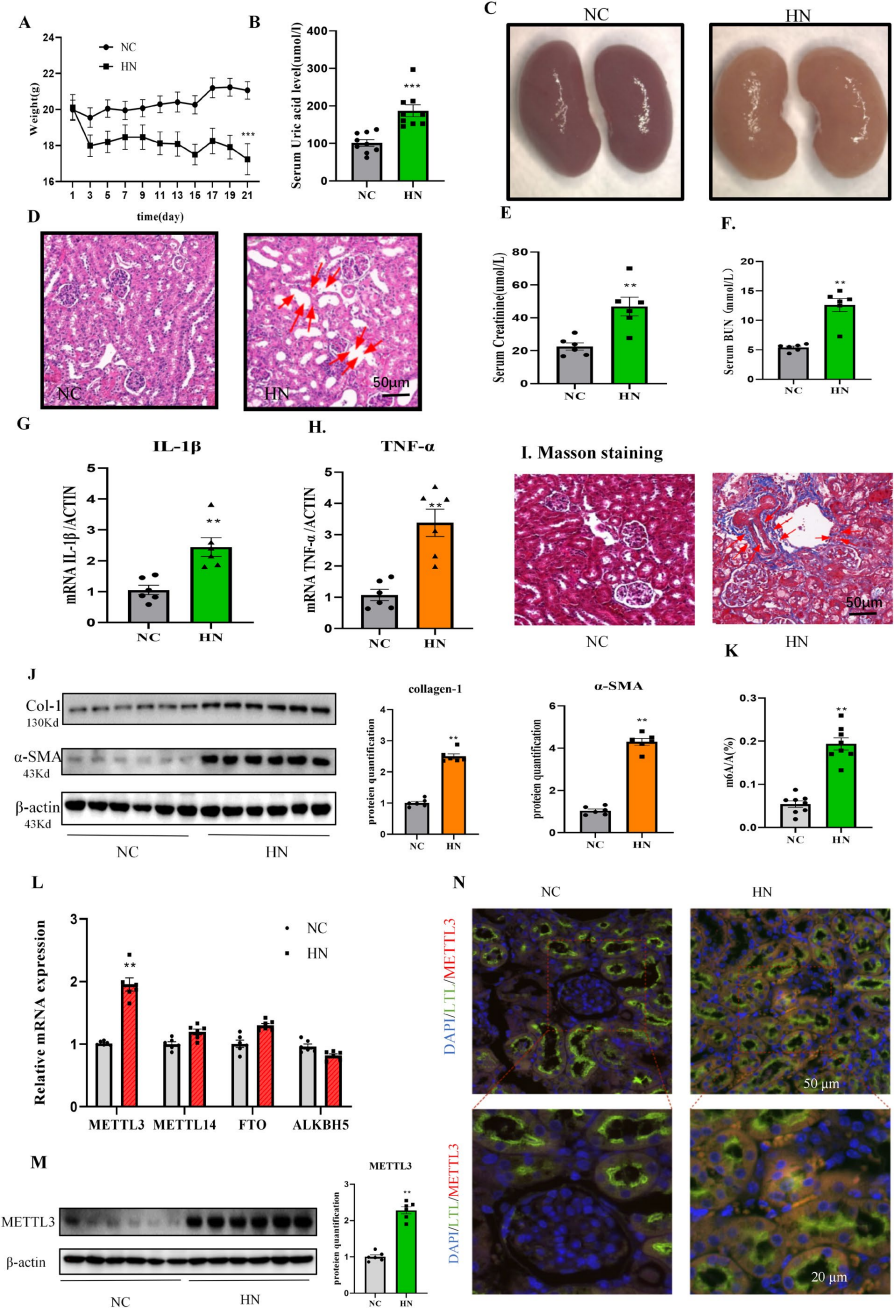
METTL3 expression are highly induced in the mouse model in response to hyperuricemia. (A) The weight of mice. (B) Serum uric acid level. (C) Kidney tissues of mice in different treatment groups. (D) HE staining of renal tissue. Scale bars = 50 μm. Data represent the mean ± S.E.M. *n* = 6 in each group. (E) Serum creatinine. (F) Blood urea nitrogen (BUN). (G‐H) Real‐time PCR of IL‐1β and TNF‐α in kidney. (I) Representative images of Masson staining in kidney slices. (J) Western blot and quantitative analysis of collagen‐1 and α‐SMA in kidney. (K) Overall m6A level detection. (L) Real‐time PCR of METTL3, METTL14, FTO, and ALKBH5 in kidney. (M) Western blot and quantitative analysis of METTL3 in kidney. (N) Immunofluorescence of METTL3 and LTL in kidney. Scale bars = 50 μm. Data represent the mean ± S.E.M. *n* = 6 in each group. **p < 0.01, ***p < 0.001 compared to the control group. Col‐1, collagen‐1; HN, hyperuricemic nephropathy; LTL, *Lotus tetragonolobus* Lectin; NC, normal control.

By day 21, mice in the potassium oxazinate + adenine feeding group showed a significant decrease in body weight compared to the normal group (Figure 1A). Serum uric acid levels in the HN group were markedly higher than those in the normal group (Figure 1B). Kidney observations revealed white, wrinkled kidneys with a bumpy surface in the HN group, while those in the normal group appeared bright red, round, and full (Figure 1C). Histological examination through HE staining demonstrated nuclear pyretosis, renal interstitial inflammatory infiltration, renal interstitial edema, and vacuole‐like degeneration in the kidneys of the HN group mice (Figure 1D), indicating renal tissue injury induced by high uric acid in mice. Serum BUN and Cr levels in the HN group were significantly elevated compared to the normal group (Figure 1E,F). Increased mRNA levels of inflammatory markers IL‐1β and TNF‐α in mouse kidneys were detected in the HN group (Figure 1G,H), indicating inflammation induced by high uric acid. Masson staining revealed prominent fibrosis in the kidneys of the HN group (Figure 1I), and western blot analysis confirmed elevated protein levels of collagen‐1 and α‐SMA, further indicating fibrotic changes in the kidneys of mice in the HN group (Figure 1J). These findings collectively demonstrate renal dysfunction, inflammation, and fibrosis induced by hyperuricemia in mice.

Our previous study has discovered that METTL3 is consistently induced in mouse models of acute kidney injury (AKI) as well as in human biopsies of AKI, demonstrating its significant association with renal inflammation and injury [13]. However, the function of METTL3 in HN is still not clear. To ascertain whether methylation or demethylation is dominant in HN, we assessed the levels of m6A methylation in the total mRNA of kidneys from HN mice. As shown in Figure 1K, the level of m6A methylation in the HN group mice was significantly higher than that in the normal group (*p* < 0.01). Using real‐time PCR technology, it was found that, compared with the normal group, the level of METTL3 was significantly increased in the HN group mice, while the levels of METTL14, FTO, and ALKBH5 showed no significant changes (Figure 1L). Furthermore, western blot analysis revealed that the protein expression of METTL3 in the kidneys of HN group mice was also significantly elevated compared to the normal group (Figure 1M, *p* < 0.01). To verify the primary renal cell type with high expression of METTL3, we performed double fluorescent staining of METTL3 and the renal tubular epithelial cell marker (Fluorescein labelled *L. tetragonolobus* Lectin, LTL). The results showed that METTL3 is mainly expressed in renal tubular epithelial cells (Figure 1N). Based on this finding, we primarily selected renal tubular epithelial cells for our in vitro studies. These results suggested that METTL3 may play an important role in the upregulation of m6A methylation levels in kidney tissues in the setting of hyperuricemia.

### 3.2 METTL3 Inhibition Alleviates Uric Acid‐Induced Inflammatory and Fibrotic Response In Vitro

**FIGURE 2:**
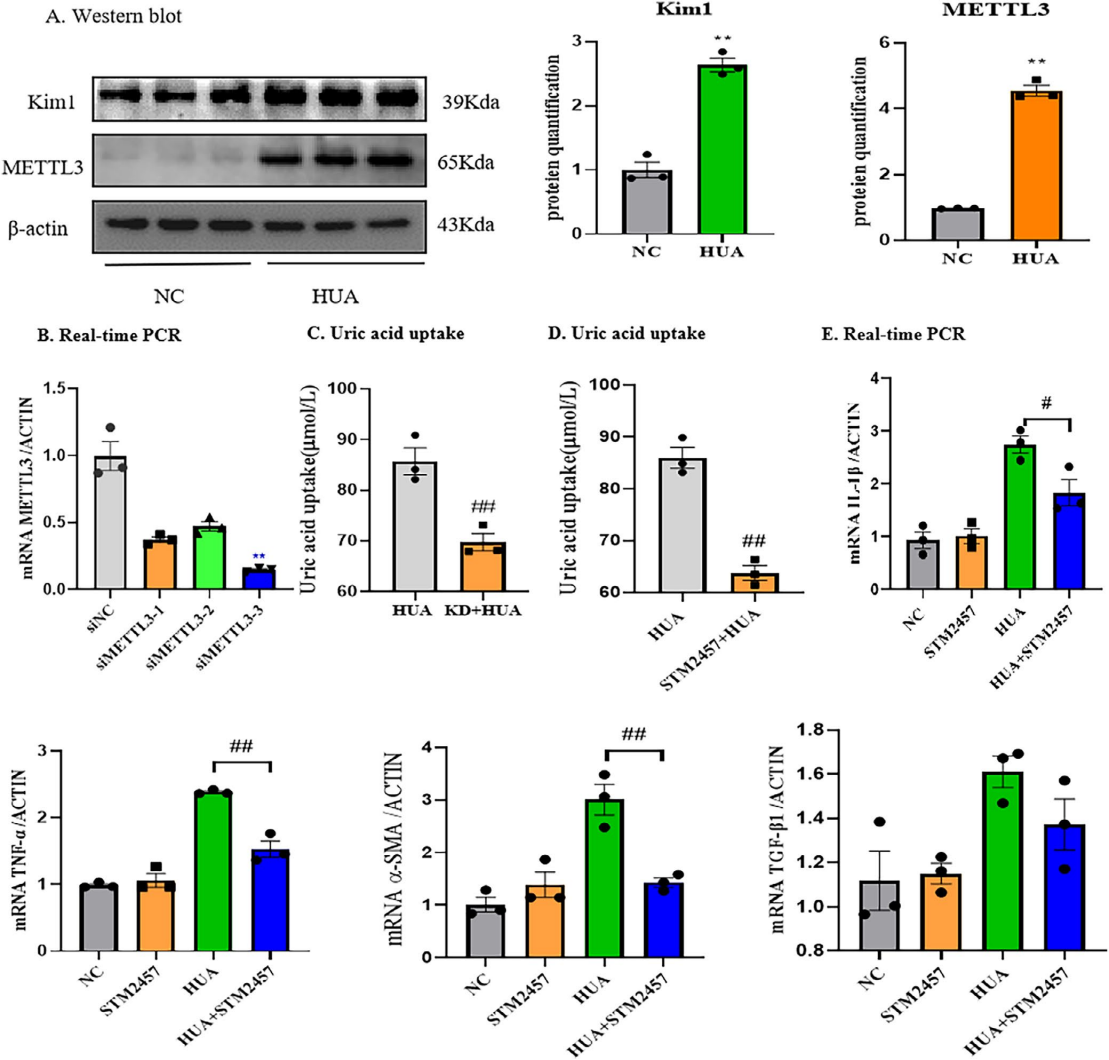
METTL3 inhibition alleviates uric acid‐induced inflammatory and fibrotic response in vitro. (A) Western blot and quantitative analysis of Kim1 and METTL3 in kidney. (B) Real‐time PCR of METTL3 (*n* = 3). (C) Uric acid uptake in METTL3 knockdown mTECs. (D) Uric acid uptake in METTL3 inhibitor treated mTECs. (E) Real‐time PCR of inflammatory factors IL‐1β and TNF‐α and fibrotic factors TGF‐β1 and α‐SMA. Data represent the mean ± S.E.M. *n* = 3 in each group. **p < 0.01 compared to the control group; ^#^ *p* < 0.05, ^##^ *p* < 0.01, compared to the HUA group. HUA, high uric acid; Kim1, kidney injury molecule‐1; KD, knockdown; NC, normal control.

To study the effects of uric acid on renal tubular epithelial cells, based on the literature, we stimulated renal tubular epithelial cells (mTECs) with a medium containing 15 mg/dL uric acid to construct a cell model [24]. We assessed the expression level of METTL3 by western blot. The results showed that the stimulation of mTECs with high levels of uric acid also led to a significant increase in the expression of METTL3 (Figure 2A). We then detected the expression of the protein kidney injury molecule‐1 (Kim‐1) (Figure 2A). The results indicated that high uric acid damaged mTECs. Next, we tried to observe the role of METTL3 on the high uric acid‐induced mTECs injury. siRNA was used to silence METTL3, and siMETTL3‐3 had the best silencing effect (Figure 2B). The result suggested that silencing METTL3 significantly inhibited the uric acid uptake by mTECs (Figure 2C). Next, STM2457 is a potent, specific, and bioavailable inhibitor of METTL3, which not only shows a high degree of specificity for METTL3 but also has no inhibitory effect on other RNA methyltransferases. Treatment with STM2457 significantly inhibited the uric acid uptake by mTECs (Figure 2D) and alleviated uric acid‐induced inflammatory factors (IL‐1β and TNF‐α) and fibrotic factors (TGF‐β1 and α‐SMA) in mTECs (Figure 2E).

### 3.3 ABCG2 Is a Direct Target of METTL3

**FIGURE 3:**
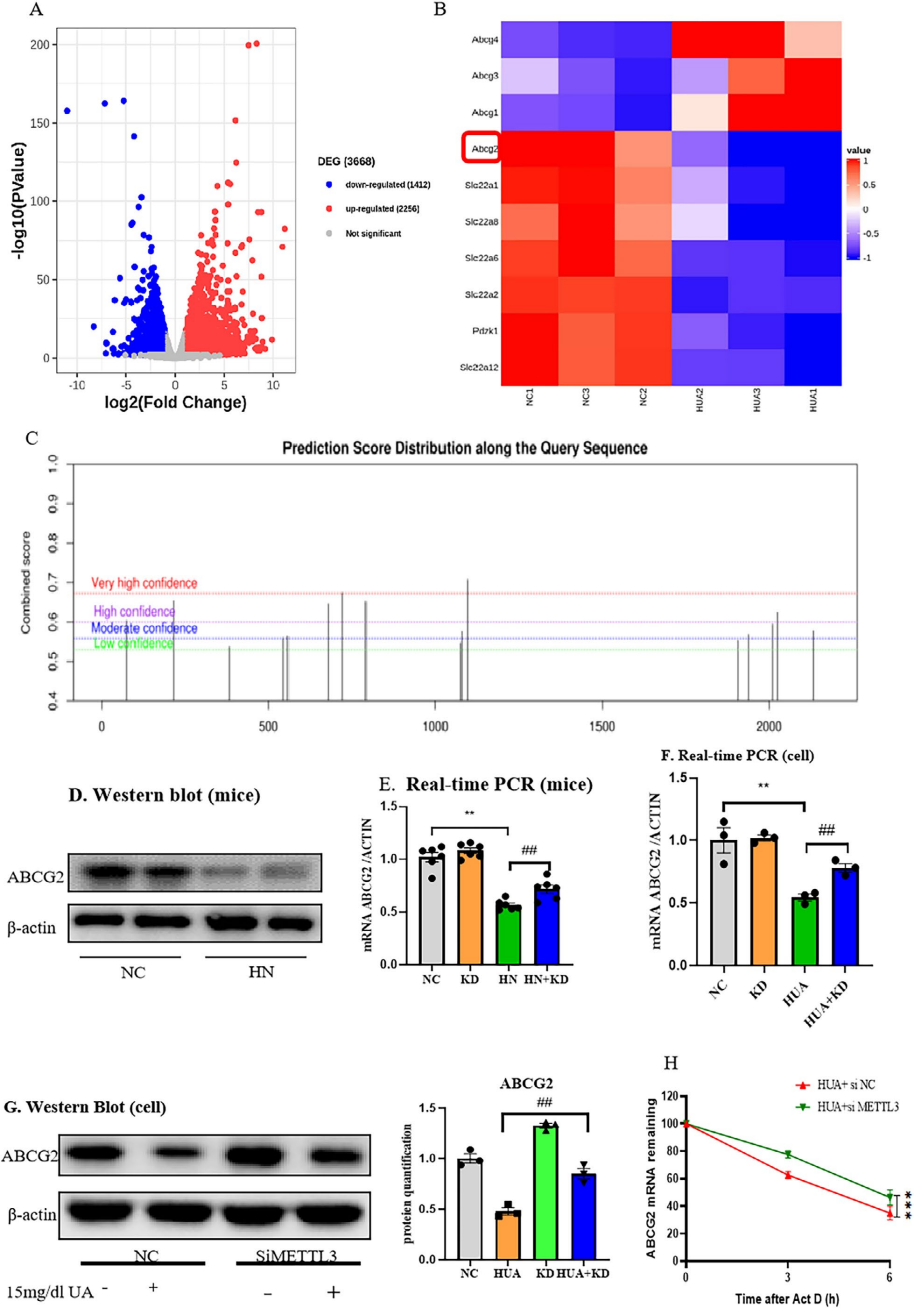
ABCG2 is a direct target of METTL3. (A, B) RNA‐seq heatmap of ABC transporters. (C) SRAMP database analysis. (D) Western blot and quantitative analysis of ABCG2 in kidney. (E) Real‐time PCR of ABCG2 in mice. (F) Real‐time PCR of ABCG2 in mTECs. (G) Western blot and quantitative analysis of ABCG2 in mTECs. (H) The actinomycin D assay shows the stability of ABCG2 in mTEC cells with METTL3 knockdown. Data represent the mean ± S.E.M. *n* = 3 in each group. **p < 0.01 compared to the control group. ^##^ *p* < 0.01, compared to the HN or HUA group. HN, hyperuricemic nephropathy; NC, normal control.

Through transcriptome sequencing of the mouse kidney tissue, it was discovered that there are 3668 sets of differential genes, of which 1412 sets of genes are downregulated in HN and 2256 sets of genes are upregulated in HN (Figure 3A). From these, we have screened out several gene sets with significant differences including ABCG2, PDZK1, slc22a2, slc22a8, and slc22a12 (Figure 3B). After querying the significantly changed gene sets from the above sequencing results through the SRAMP database, it was found that ABCG2 not only had significant variance but also possessed several sensitive m6A modification sites (Figure 3C). ABCG2, as an important transporter protein, plays a significant role in uric acid excretion. Real‐time fluorescent quantitative PCR and western blot results showed that compared to the normal group, the expression of ABCG2 in the kidneys of the HN mice was significantly reduced (Figure 3D,E).

Based on the above experimental results, we hypothesize that HN may influence the expression of ABCG2 and its function, thus affecting the excretion and absorption of uric acid, possibly through METTL3‐mediated m6A methylation of ABCG2. To test this hypothesis, we will use the METTL3 siRNA to silence METTL3 and then observe the changes in ABCG2 protein expression. Silencing METTL3 in mice can improve the decline of the ABCG2 mRNA level caused by hyperuricemia (Figure 3E); consistent results have been observed in vitro (Figure 3F). mTEC cells were stimulated with 15 mg/dL UA. Protein extraction was performed, and the level of ABCG2 was detected. The results showed that silencing METTL3 significantly inhibited the decrease in ABCG2 protein expression (Figure 3G). We detected a shortened mRNA half‐life of ABCG2 in METTL3‐deficient mTEC cells, suggesting that m6A modification of ABCG2 reduces its mRNA stability (Figure 3H).

### 3.4 Inhibition of METTL3 Upregulated ABCG2 Expression in mTECs In Vitro

**FIGURE 4:**
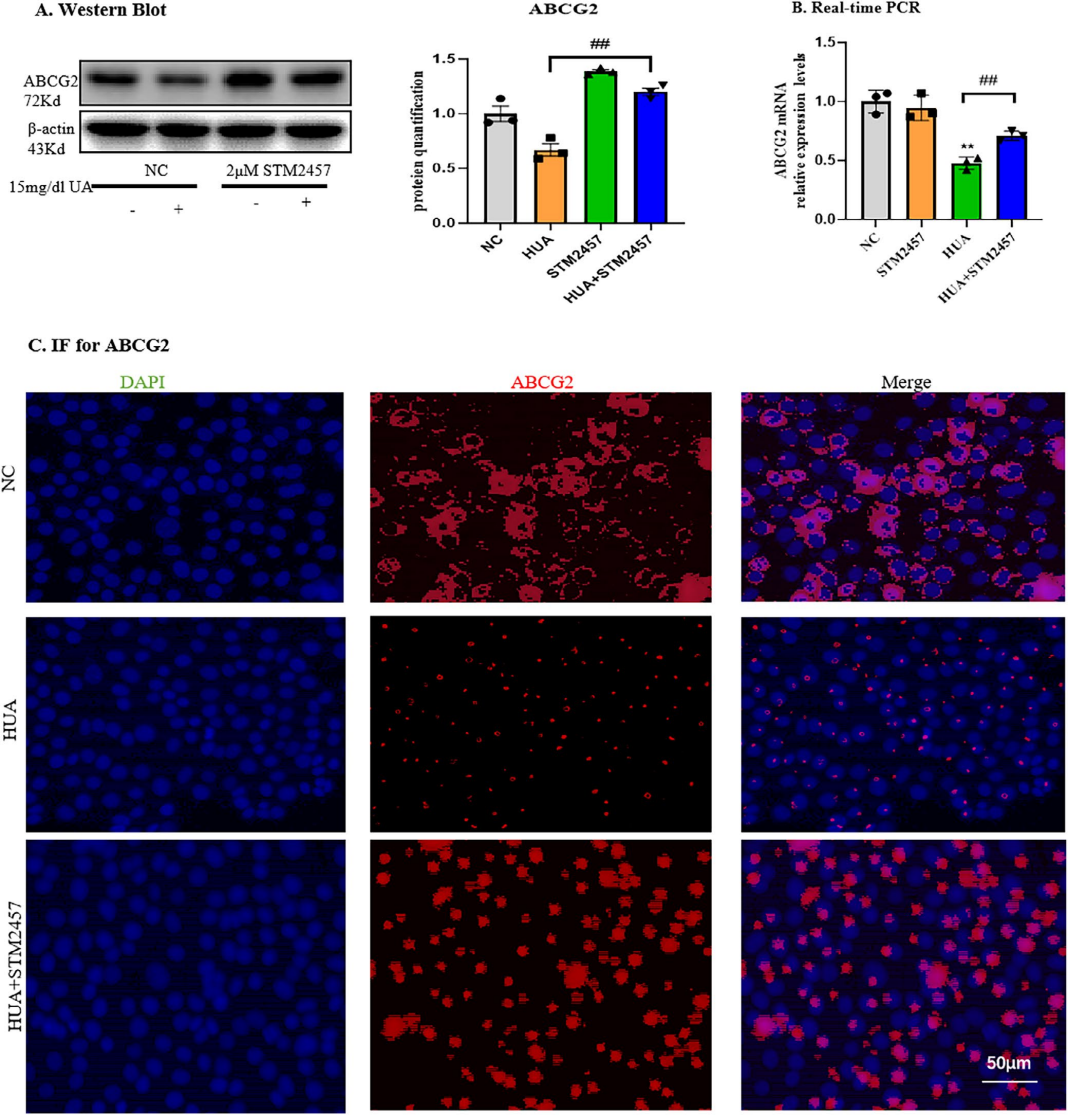
Inhibition of METTL3 increased expressions of ABCG2. (A) Western blot and quantitative analysis of ABCG2 in mTECs. (B) Real‐time PCR of ABCG2 in mTECs. (D) Immunofluorescence assay of ABCG2 in mTECs. Scale bars = 50 μm. Data represent the mean ± S.E.M. *n* = 3 in each group in vitro. ^##^ *p* < 0.01, compared to the HUA group. NC, normal control.

Next, the role of the METTL3‐specific inhibitor STM2457 on ABCG2 expression in mTECs was observed. The result showed that STM2457 effectively mitigated or even reversed the decline of ABCG2 (Figure 4A), which was confirmed by real‐time PCR (Figure 4B). Immunofluorescence assay also confirmed that STM2457 significantly upregulated the expression of ABCG2 in the mTECs (Figure 4C).

### 3.5 IGF2BP2 Reduces ABCG2 mRNA Stability in an m6A‐Dependent Manner

**FIGURE 5:**
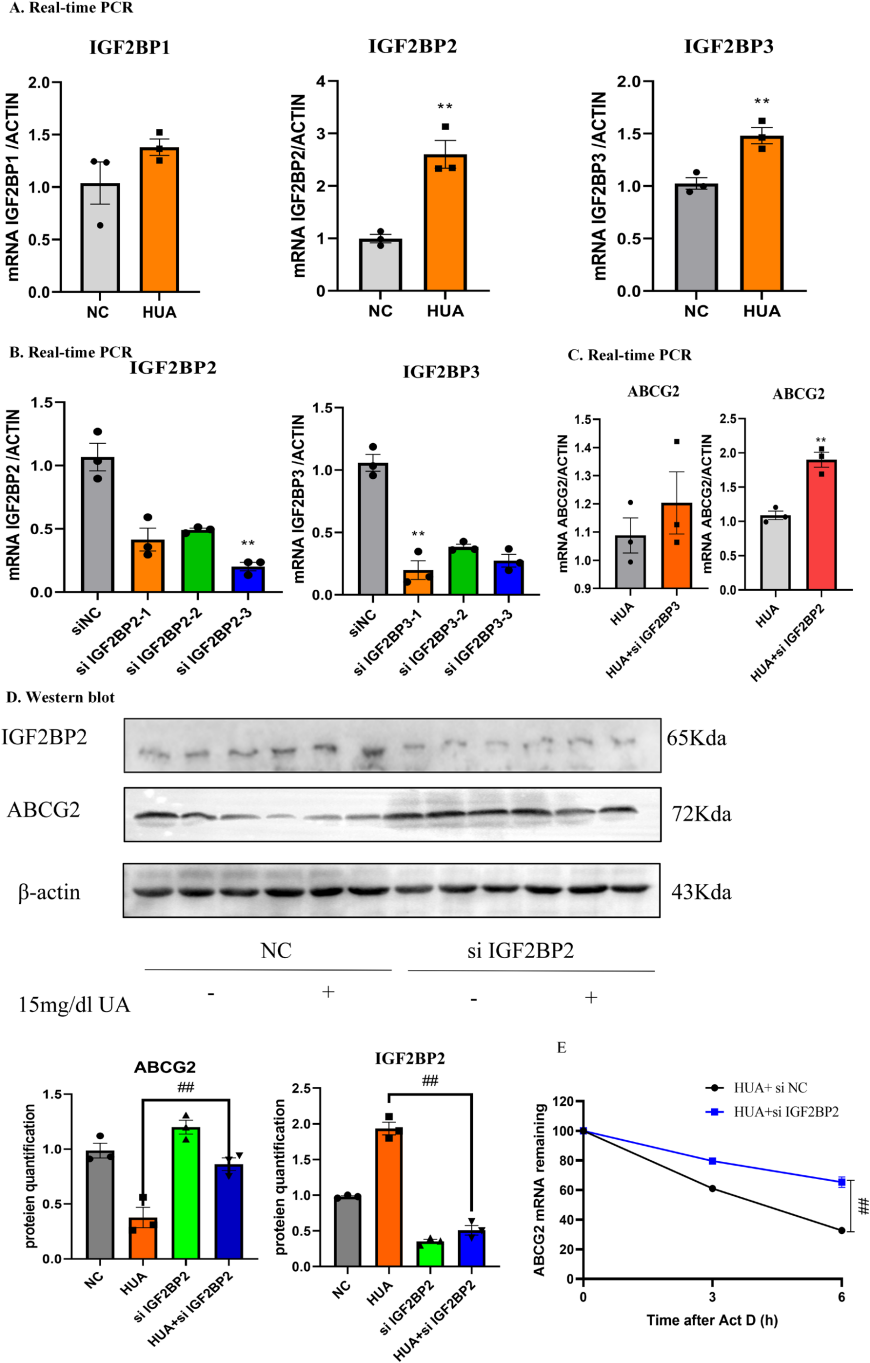
IGF2BP2 reduces ABCG2 mRNA stability in an m6A‐dependent manner. (A) Real‐time PCR analysis of IGF2BP1, IGF2BP2, and IGF2BP3 in mTEC cells. (B) Real‐time PCR analysis of IGF2BP2 and IGF2BP3 in mTEC cells transfected with si‐IGF2BP2 or si‐IGF2BP3. (C) Real‐time PCR analysis of ABCG2 in mTEC cells transfected with si‐IGF2BP2 or si‐IGF2BP3. (D) Western blot and quantitative analysis of IGF2BP2 and ABCG2 in mTECs. (E) The actinomycin D assay shows the stability of ABCG2 in mTEC cells with IGF2BP2 knockdown. Data represent the mean ± S.E.M. *n* = 3 in each group. **p < 0.01 compared to the control group. ^##^ *p* < 0.01 compared to the HUA group. HUA, high uric acid; NC, normal control.

Considering that IGF2BP (insulin‐like growth factor 2 binding protein) family members play a key role in mediating the stability and translation of m6A‐modified mRNAs, we assessed the involvement of IGF2BP1/2/3 in ABCG2 mRNA stabilisation. At first, we measured the mRNA levels of IGF2BP1, IGF2BP2, and IGF2BP3. The results showed that the mRNA levels of both IGF2BP2 and IGF2BP3 were upregulated (Figure 5A). Therefore, three specific siRNAs were designed against each target of IGF2BP2 and IGF2BP3, and the knockdown efficiency of these constructs was confirmed (Figure 5B). We found that silencing IGF2BP2 markedly upregulated ABCG2 mRNA expression, but the knockdown of IGF2BP3 had a limited effect (Figure 5C). In addition, Western blot analysis indicated that IGF2BP2 knockdown also increased ABCG2 protein expression (Figure 5D). An RNA stability assay showed that IGF2BP2 knockdown after actinomycin D (5 g/mL) treatment increased the stability of ABCG2 mRNA, indicating that IGF2BP2 has a critical role in the maintenance of ABCG2 mRNA stability (Figure 5E).

### 3.6 AAV9‐Mediated Silencing of METTL3 Protects Against Renal Fibrosis in HN

**FIGURE 6:**
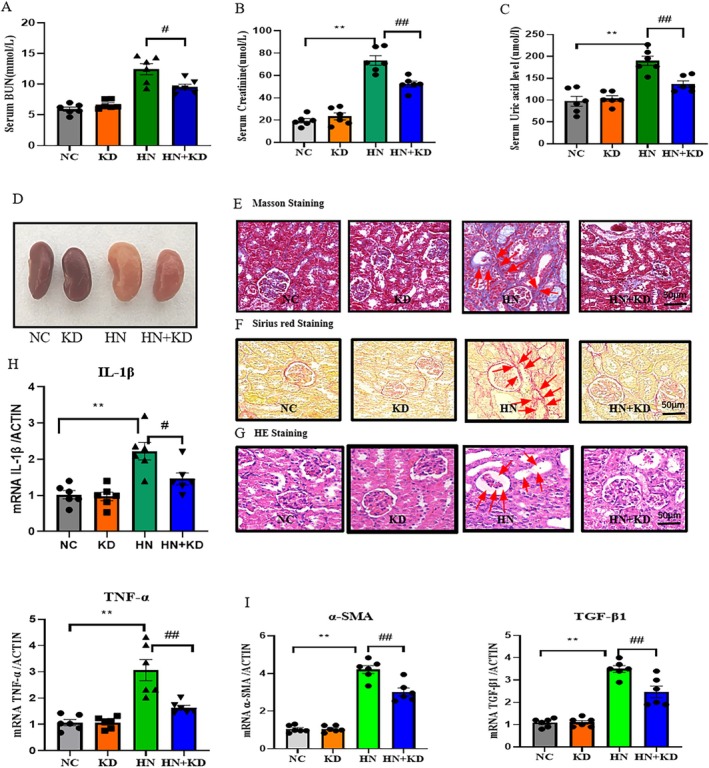
AAV9‐mediated silencing of METTL3 protects against renal fibrosis in HN. (A) Blood urea nitrogen (BUN). (B) Serum creatinine. (C) Serum uric acid level. (D) Kidney tissues of mice in different treatment groups. (E) Representative images of Masson staining in kidney slices. (F) Representative images of Sirius red staining in kidney slices. (G) Representative images of HE staining in kidney slices. (H) Real‐time PCR of inflammatory factors IL‐1β and TNF‐α. (I) Real‐time PCR of fibrotic factors TGF‐β1 and α‐SMA. **p < 0.01 compared to the control group. Data represent the mean ± S.E.M. *n* = 6 in each group. ^#^ *p* < 0.05, ^##^ *p* < 0.01, compared to the HN group. HN, hyperuricemic nephropathy; KD, knockdown; NC, normal control.

To determine the therapeutic potential of METTL3 in HN mouse models, we silenced METTL3 in mice using an AAV9‐packaged METTL3 knockdown plasmid. We found that the disruption of METTL3 decreased serum BUN and Cr concentrations (Figure 6A,B). Furthermore, METTL3 silencing decreased serum uric acid levels (Figure 6C). Histological analysis confirmed the restoration of kidney morphology, reduced inflammation, and fibrosis, confirmed by H&E staining, Sirius red staining, and Masson staining (Figure 6D–G). Real‐time PCR analysis further validated decreased mRNA expression of inflammatory markers (IL‐1β and TNF‐α) and fibrosis factors (α‐SMA and TGF‐β1) (Figure 6H,I).

## 4 Discussion

Traditionally, hyperuricemia has been believed to cause kidney disease through the deposition of uric acid crystals in nephron collecting ducts [25]. Recent studies have revealed multiple mechanisms contributing to HN, including activation of the angiotensin system, endothelial dysfunction, enhancement of the inflammatory response, oxidative stress, and apoptosis of kidney residential cells [26, 27]. However, few studies have focused on the role of m6A modifications in HN. Here, we linked m6A modifications to HN. The m6A level and METTL3 expression were highly induced in TECs in response to high uric acid stimuli. METTL3 promoted renal inflammation and fibrosis by downregulating ABCG2 expression through decreasing its RNA stability in IGF2BP2‐dependent mechanisms. Silencing METTL3 alleviated uric acid‐driven renal inflammation and fibrosis in AAV9‐mediated METTL3 silencing mice and METTL3 knockdown mTECs. We found that treatment with STM2457 or with AAV9‐mediated METTL3 silencing protected against renal fibrosis and inflammation. These results collectively showed that the METTL3/ABCG2 axis is a promising therapeutic target for HN.

Recent experimental studies have illuminated that HN is a common clinical complication of hyperuricemia. High serum uric acid can trigger renal inflammation and lead to renal fibrosis [27, 28]. While the secretion and reabsorption of uric acid primarily occur through renal tubules [29], high blood uric acid not only stimulates renal tubular epithelial cells but also prompts the release of various chemokines, recruiting inflammatory cells and increasing inflammation, ultimately contributing to injury [30]. Additionally, studies have reported that elevated uric acid can stimulate epithelial cells from renal tubules to undergo epithelial‐mesenchymal transitions (EMT), leading to renal fibrosis [31]. Thus, tubular epithelial cells likely play a pivotal role in renal dysfunction, inflammation, and fibrosis mediated by high uric acid, although the specific mechanisms warrant further exploration.

The above findings indicate that the imbalance of uric acid metabolism causing hyperuricemia can lead to kidney inflammation and fibrosis. It has been reported that epigenetic modification, particularly RNA methylation, plays a key role in the regulation of gene expression, and it has been found to be associated with the pathophysiology of various kidney diseases [13, 32]. In this study, it was observed that the m6A methylation level was significantly increased in the HN group due in part to an increase in the METTL3 levels in the kidneys. It was also found that METTL3 was localised in renal tubular epithelial cells. Further in vitro experiments using mTECs exposed to high uric acid levels revealed inflammation, fibrotic response, and increased METTL3 levels in these cells. We also confirmed the importance of METTL3 in renal fibrosis by using AAV9‐mediated METTL3 silencing mice with hyperuricemia or a specific METTL3 inhibitor STM2457. These findings suggest that hyperuricemia causes renal dysfunction, inflammation, and fibrosis in mice, and METTL3 plays significant roles in this process.

Next, the detailed mechanisms by which METTL3 promotes renal inflammation were determined. RNA‐seq analysis was performed to identify gene expression differences between the normal and HN groups, and the data analysis revealed that ABCG2 was one of the genes with significant differences and multiple sensitive m6A modification sites. We confirmed the regulation of METTL3 on ABCG2 by using METTL3 silencing or inhibition. ABCG2, as a uric acid transporter, is expressed on the lumen‐facing apical membrane of renal epithelial cells and exits uric acid from the cells into the tubular lumen across the brush‐border membrane in the kidney [18]. While ABCG2 dysfunction is a common mechanism of hyperuricemia [33]. However, the function of ABCG2 mediated by METTL3 in the development of HN was unclear. We found that ABCG2 is reduced in HN and its upregulation attenuated renal inflammation and fibrosis.

The fate of target transcripts typically depends on the specific recognition of m6A readers, such as members of the IGF2BP family, including IGF2BP1, IGF2BP2, and IGF2BP3, which serve as one of the most common m6A readers for recognising m6A modifications [13]. In our study, we found that IGF2BP2 was significantly upregulated in high uric acid‐treated mTECs. IGF2BP2 is composed of two RRM domains and four KH domains and plays regulatory roles in post‐transcriptional processes through the IGF2BP2‐RNA complex [34]. Many studies demonstrate that IGF2BP2 can promote target expression by enhancing mRNA stability [13], while Pan et al. reported that IGF2BP2 could destabilise its target mRNA, thus indicating the complexity of the regulatory role of IGF2BP2 [35]. In our study, we got the consistent result. We found that IGF2BP2 could inhibit the stability of ABCG2 mRNA. We confirmed that IGF2BP2, instead of IGF2BP1/3, plays a key role in the ABCG2 mRNA stability under the control of METTL3.

## 5 Conclusions

Our results showed that METTL3 expression is significantly induced in mTECs in response to high levels of uric acid, and METTL3 promotes renal inflammation and injury by decreasing ABCG2 m6A RNA methylation through decreasing its RNA stability in IGF2BP2‐dependent mechanisms. Our work sheds light on the poorly understood molecular mechanisms by which m6A contributes to HN and suggests that targeting the METTL3/ABCG2 axis is a potential strategy against HN. Certainly, further investigations are needed to explore the detailed molecular mechanisms underlying this association and potential therapeutic strategies to target these pathways in the treatment of HN and related kidney diseases.

## Author Contributions

**Juan Jin:** data curation (equal), resources (equal), software (equal), supervision (equal), validation (equal), visualization (equal). **Tong Zu:** data curation (equal), funding acquisition (equal), methodology (equal), supervision (equal), writing – original draft (equal). **Hang Yang:** data curation (equal), methodology (equal). **Yue Yu:** methodology (equal). **Kuo Zhang:** methodology (equal). **Jie Wang:** data curation (equal). **Xiuxiu Song:** investigation (equal). **Jie Ying:** conceptualization (equal). **Xian Wang:** formal analysis (equal), investigation (equal). **Yaru Yang:** funding acquisition (equal), supervision (equal), writing – review and editing (equal). **Shuangjian Li:** investigation (equal).

## Disclosure

Declaration of Generative AI in Scientific Writing: We did not use artificial intelligence during the scientific writing.

## Conflicts of Interest

The authors declare no conflicts of interest.

## Data Availability

Data will be made available on request.
